# Evolution of plant RNA polymerase IV/V genes: evidence of subneofunctionalization of duplicated *NRPD2/NRPE2*-like paralogs in *Viola *(Violaceae)

**DOI:** 10.1186/1471-2148-10-45

**Published:** 2010-02-16

**Authors:** Thomas Marcussen, Bengt Oxelman, Anna Skog, Kjetill S Jakobsen

**Affiliations:** 1 Centre for Ecological and Evolutionary Synthesis (CEES), Department of Biology, University of Oslo, 0316 Oslo, Norway; 2 Department of Plant and Environmental Sciences, University of Gothenburg, SE-40530 Göteborg, Sweden

## Abstract

**Background:**

DNA-dependent RNA polymerase IV and V (Pol IV and V) are multi-subunit enzymes occurring in plants. The origin of Pol V, specific to angiosperms, from Pol IV, which is present in all land plants, is linked to the duplication of the gene encoding the largest subunit and the subsequent subneofunctionalization of the two paralogs (*NRPD1 *and *NRPE1*). Additional duplication of the second-largest subunit, *NRPD2/NRPE2*, has happened independently in at least some eudicot lineages, but its paralogs are often subject to concerted evolution and gene death and little is known about their evolution nor their affinity with Pol IV and Pol V.

**Results:**

We sequenced a ~1500 bp *NRPD2/E2*-like fragment from 18 *Viola *species, mostly paleopolyploids, and 6 non-*Viola *Violaceae species. Incongruence between the *NRPD2/E2*-like gene phylogeny and species phylogeny indicates a first duplication of *NRPD2 *relatively basally in Violaceae, with subsequent sorting of paralogs in the descendants, followed by a second duplication in the common ancestor of *Viola *and *Allexis*. In *Viola*, the mutation pattern suggested (sub-) neofunctionalization of the two *NRPD2/E2*-like paralogs, *NRPD2/E2-a *and *NRPD2/E2-b*. The *d*_*N*_/*d*_*S *_ratios indicated that a 54 bp region exerted strong positive selection for both paralogs immediately following duplication. This 54 bp region encodes a domain that is involved in the binding of the Nrpd2 subunit with other Pol IV/V subunits, and may be important for correct recognition of subunits specific to Pol IV and Pol V. Across all *Viola *taxa 73 *NRPD2/E2*-like sequences were obtained, of which 23 (32%) were putative pseudogenes - all occurring in polyploids. The *NRPD2 *duplication was conserved in all lineages except the diploid MELVIO clade, in which *NRPD2/E2-b *was lost, and its allopolyploid derivates from hybridization with the CHAM clade, section *Viola *and section *Melanium*, in which *NRPD2/E2-a *occurred in multiple copies while *NRPD2/E2-b *paralogs were either absent or pseudogenized.

**Conclusions:**

Following the relatively recent split of Pol IV and Pol V, our data indicate that these two multi-subunit enzymes are still in the process of specialization and each acquiring fully subfunctionalized copies of their subunit genes. Even after specialization, the *NRPD2/E2*-like paralogs are prone to pseudogenization and gene conversion and *NRPD2 *and *NRPE2 *copy number is a highly dynamic process modulated by allopolyploidy and gene death.

## Background

Eukaryotes normally possess three nuclear DNA-dependent RNA polymerases (Pols), Pol I-III, functionally specialized for synthesis of different types of RNA and thus essential for viability. The Pol holoenzymes consist of about 12 subunits, of which the two largest are tightly bound and together constitute the catalytic seat of the enzyme and are generally polymerase-type specific [1-4]. Angiosperms (flowering plants) are unique in possessing two additional RNA polymerases that are not essential for viability, Pol IV and Pol V (previously called Pol IVa and IVb, or RNAP IVa and IVb). They are functionally distinct, with Pol IV being required for 24 nt siRNA production and Pol V for siRNA-mediated gene silencing of transposons and other repeated elements [5].

The subunit nomenclature of nuclear RNA polymerases has varied among research groups and organisms, and is often in conflict with names for unrelated genes. In the following, we have therefore adopted the 4-letter gene names registered with The *Arabidopsis *Information Resource. By convention the largest subunits of Pol I, II, III, IV and V are Nrpa1, Nrpb1, Nrpc1, Nrpd1 and Nrpe1 respectively, and the genes encoding these are *NRPA1*, *NRPB1*, *NRPC1*, *NRPD1 *and *NRPE1*, respectively. Likewise, the genes encoding the second-largest subunits of the five polymerases are designated *NRPA2*, *NRPB2*, *NRPC2*, *NRPD2 *and *NRPE2*, respectively.

The genes encoding the largest and second-largest subunits of Pol IV, *NRPD1 *and *NRPD2 *respectively, originated by independent duplication of their Pol II homologs, *NRPB1 *and *NRPB2*. The *NRPB1*/*NRPD1 *duplication is shared by both charophytes and embryophytes while the *NRPB2*/*NRPD2 *duplication is found only in embryophytes [3]. While Pol IV is found in all plants, Pol V appears to exist only in angiosperms (flowering plants) following duplication of, at least, the largest subunit gene (*NRPD1/NRPE1*) basally in this lineage [3]. A recent study in the eudicot angiosperm *Arabidopsis thaliana *confirms the close relationship of Pol IV and Pol V with Pol II and shows that many of their 12 subunits are shared among these three RNA polymerases [1]. Nevertheless, 4 subunits of Pol IV and 6 subunits of Pol V are distinct from their Pol II paralogs, and Pol IV and Pol V differ in 4 subunits. Interestingly, 3 duplicated Pol IV/V genes (third, seventh and nineth largest subunits) appear to be incompletely subfunctionalized with respect to Pol IV and Pol V. These have a higher sequence similarity than the fully specialized gene pairs (e.g. *NRPD1/NRPE1*) and are presumably derived from more recent duplication events.

Following the duplication and specialization of the Pol IV/V largest subunit genes (*NRPD1*/*NRPE1*) in angiosperms, duplication of the second-largest subunit genes (*NRPD2/E2*) seems comparatively rare. *NRPD2/E2 *is apparently a singleton in monocots (*Oryza*, *Zea*) as well as in several families of eudicots, e.g., Aceraceae (*Acer*), Asteraceae (*Carthamus*), Lamiaceae (*Galeopsis*; Brysting AK, unpublished), Myrtaceae (*Myrtus*), Solanaceae (*Solanum*) and Vitaceae (*Vitis*) [3,6-8]. A few eudicot lineages, however, possess duplicate *NRPD2/E2 *copies, e.g., Brassicaceae (*Arabidopsis*; but only one paralog is expressed), Caprifoliaceae (*Lonicera*), Celastraceae (*Maytenus*), Euphorbiaceae (*Manihot*), Salicaceae (*Populus*; but only one paralog is expressed), Caryophyllaceae (*Silene *and many other genera) and Violaceae (*Viola *and *Allexis*; herein) [6,9-11]. This indicates that duplicated *NRPD2/E2 *genes may in fact be a common feature in eudicots. It is clear that the *NRPD2/E2 *duplications have occurred independently in these lineages, and that they are also frequently lost, with sorting among lineages as a common result [10].

The mechanisms behind gene duplication are well known in eukaryotes and in plants [e.g., [12]]. While it is clear that by far the most likely fate of a duplicate gene is gene death [7,13,14], mechanisms accounting for the duplications being retained in the genome have been, until recently, less well understood [15]. Duplicate genes may be preserved by a neutral mechanism in which each paralog accumulates loss-of-function mutations (degeneration) that are complemented by the other copy. Such mutations can happen either at the regulatory level, causing the paralogs to diverge in pattern of expression (duplication-degeneration-complementation (DDC) [16]), or at the product level, causing the paralogs to diverge in function (subfunctionalization [17]). Furthermore, either mechanism can eliminate possible structural trade-offs imposed by different functions performed by a multifunctional gene [18], by unlinking these functions. These mechanisms can thus be regarded as prerequisites for the ability of duplicate genes to specialize and acquire new functions (subneofunctionalization [19]). Regulatory and functional subfunctionalization are both well-documented in gene families, in eukaryotes in general as well as in plants [e.g., [15,20-23]].

The RNA polymerase subunit encoded by *NRPD2/E2*, Nrpd2/Nrpe2, has a discrete double function in angiosperms, assembling either with Pol IV or with Pol V. A duplication of this gene might have been preserved if the two paralogs underwent subfunctionalization with respect to Pol type, and would have required some degree of co-evolution of co-assembling subunits.

**Figure 1:**
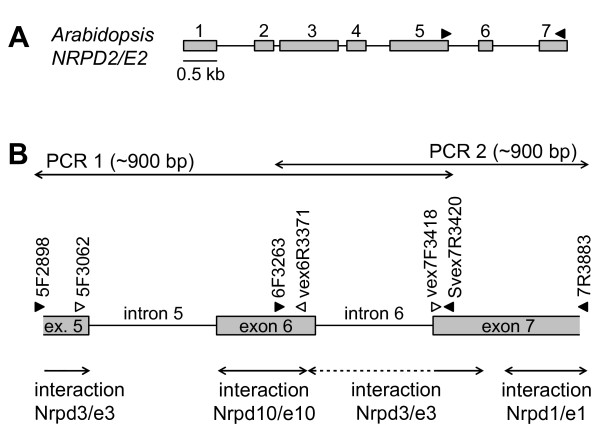
**Sequence characteristics of *NRPD2/E2***. A. The corresponding localization in *Arabidopsis NRPD2/E2 *of the region amplified for Violaceae. B. Specifications for the region in Violaceae. Filled triangles denote PCR primers used to amplify the two standard PCR regions, and open triangles denote primers used to amplify shorter stretches if PCRs with the standard primers repeatedly failed. *NRPD2/E2 *domains involved in recognition and binding to other Pol IV/V subunits are indicated, based on findings in yeast Pol II [2].

In this study we have investigated the evolution of *NRPD2/E2*-like genes within the Violaceae (Malpighiales), with particular reference to the genus *Viola *(Figure 1). This gene occurs in a single copy in most genera of the family but it is duplicated in others (*Allexis *and *Viola*). In a similar system within tribe *Sileneae *of the Caryophyllaceae (Caryophyllales), concerted evolution was found to be prominent among *NRPD2/E2 *paralogs [10]. In that study, however, only intron 6 was investigated. In order to be able to examine possible neofunctionalization among *NRPD2/E2*-like duplicants in the Violaceae system, we have expanded this range to include also the flanking exons of intron 6.

The Violaceae consist of some 900 species in 23 mostly tropical genera [24]. Their relationships have recently been examined in a phylogenetic study based on plastid and nuclear ribosomal gene DNA sequences [25]. With more than 500 species, *Viola *is the largest genus of the Violaceae and the only one widely distributed in the northern hemisphere [26]. Based on chromosome counts [e.g., [27]] and isozyme expression data [28,29] it can be estimated that roughly two thirds of *Viola *species belong to paleopolyploid lineages having secondary base numbers ranging from *x *= 10 to *x *= 27 or higher. "True" diploids are known only from two sections, *Andinium *(*x *= 7) from South America [30] and *Chamaemelanium *(*x *= 6) which is mainly northern amphi-Pacific [e.g., [31]]. Tentative genus phylogenies have been based on rRNA Internal Transcribed Spacer (ITS) sequence data in several studies [e.g., [26,32]] but this marker has proven of no use for recovering any of these polyploid relationships [e.g., [33]]. The genus phylogeny is currently being re-examined using low-copy nuclear genes (Marcussen, Oxelman, Jakobsen, unpublished data).

In *Arabidopsis *five of the 12 genes associated with Pol IV/V have been duplicated, apparently independently, and have undergone subfunctionalization with respect to Pol IV and V [cf. [1]]. For *NRPD2/E2 *in eudicots, available sequence information suggests numerous independent duplications and that these paralogs are often subject to concerted evolution and gene death [10]. In this study, we elucidate the origin of duplication of the *NRPD2/E2*-like genes within the Violaceae and aspects of its evolution and phylogeny within *Viola*. Polyploidy, which is known to be a major evolutionary process in *Viola*, could be thought to interact with a nascent gene family such as *NRPD2/E2*. For instance, could redundancy resulting from polyploidy destabilize the incipient differentiation of the two paralogs, *NRPD2/E2-a *and *NRPD2/E2-b*, or could the occasional loss of primary duplication be compensated for by secondary duplications resulting from polyploidy? The immediate consequence of gene duplication is redundancy, which will generally lead to loss or pseudogenization of one paralog unless the paralogs become subfunctionalized or neofunctionalized. Positive selection can be taken as evidence of neofunctionalization. It is therefore of relevance to detect to what degree positive selection has acted on duplicated *NRPD2/E2*-like paralogs within the Violaceae, and if it has, at which sites and on which phylogenetic branches.

## Results

### Assignment and naming of *NRPD2/E2*-like homologs in Violaceae

*NRPD2/E2-a *and *NRPD2/E2-b *are arbitrary labels that denote the two paralogs found in *Viola *and *Allexis*. They do not reflect orthology to duplicated *NRPD2/E2 *loci outside of Violaceae, and do not imply that the respective binding specificities of the paralogs to Pol IV and Pol V are known. Appended digits to the sequence name separate homoeologs of a paralog within a single specimen (e.g., *banksii_B2 *refers to homoeolog 2 of *NRPD2/E2-b *in *V*. *banksii*).

### *NRPD2/E2*-like homologs in Violaceae

GenBank sequence data for the Malpighiales demonstrate duplicate copies of *NRPD2/E2 *in both *Manihot esculenta *(CK652029, DV448133) and *Populus trichocarpa *(e.g., DT509274, CV227572) but not in *Euphorbia esula *(DV145650). In *Manihot *both paralogs are potentially functional but in *Populus *one paralog (CV227572) is characterized by frameshift and non-synonymous mutations not reconcilable with *NRPD2/E2 *activity. The two copies found in *Manihot*, *Populus *and *Viola *are not orthologous to each other (not shown). We obtained and analyzed sequence information from six non-*Viola *Violaceae taxa (Table 1). These sequences were aligned to exon (mRNA) sequences from GenBank of *Euphorbia*, *Manihot *and *Populus*. Outside *Viola*, we found singleton *NRPD2/E2 *in all of *Anchietea parvifolia*, *Corynostylis arborea*, *Cubelium concolor *(= *Hybanthus concolor*), *Hybanthus enneaspermus *and *Rinorea ilicifolia*. Like *Viola*, *Allexis batangae *had duplicated *NRPD2/E2 *genes, but only the *NRPD2/E2-b *paralog was putatively functional; its *NRPD2/E2-a *paralog was a pseudogene that contained three frameshift mutations and stop codons in all three reading frames.

**Table 1:** Material and gene sequences used

Species	Taxonomic group (base chromosome number)	2*n*	*x*	GenBank accession ID	Voucher ID
*Viola congesta*	sect. *Andinium *(*x *= 7)	--	2*x*	a: GU289564; b: GU289615	Marcussen 641 (O)

*Viola biflora*	sect. *Chamaemelanium *(*x *= 6)	2*n *= 12	2*x*	a: GU289574; b: GU289625	Marcussen 775 (O)

*Viola brevistipulata*	sect. *Chamaemelanium *(*x *= 6)	2*n *= 12	2*x*	a: GU289575; b: GU289626, GU289627	Marcussen 803 (O)

*Viola canadensis*	sect. *Chamaemelanium *(*x *= 6)	2*n *= 12, 24	4*x*	a^a^: GU289576; b^a^: GU289637	Marcussen 802 (O)

*Viola nuttallii*	sect. *Chamaemelanium *(*x *= 6)	2*n *= 24	4*x*	a: GU289577, GU289578, GU289579; b: GU289628, GU289629	Marcussen 801 (O)

*Viola pubescens*	sect. *Chamaemelanium *(*x *= 6)	2*n *= 12	2*x*	a: GU289580; b: GU289630	Marcussen 637 (O)

*Viola maculata*	sect. *Chilenium*	--	8*x*	a: GU289570, GU289571, GU289572, GU289573; b: GU289616, GU289617, (GU289618^b^), GU289619	Marcussen 804 (O)

*Viola banksii*	sect. *Erpetion*	--	8*x*-10*x*	a: GU289565, GU289566, GU289567^c^, GU289568^c^, (GU289569^b^); b: (GU289620^c^), GU289621, GU289622^b^, GU289623, GU289624	Marcussen 630 (O)

*Viola bicolor*	sect. *Melanium*	2*n *= 34	12*x*?	a: GU289603, GU289604, GU289605^c^, GU289606^b^, GU289607^c^, GU289608^b^	Marcussen 743 (O)

*Viola calcarata*	sect. *Melanium*	2*n *= 20	12*x*?	a: GU289609, GU289610, GU289611, GU289612^*c*^, GU289613, GU289614	Marcussen 672 (O)

*Viola dirimliensis*	sect. *Melanium*	2*n *= 8	8*x*?	a: GU289599, GU289600, GU289601, GU289602^c^	Marcussen 650 (O)

*Viola epipsila*	sect. *Viola *(*x *= 10, 12)	2*n *= 24	4*x*	a: GU289587, GU289588; b: GU289635^b^	Marcussen 661 (O)

*Viola hirta*	sect. *Viola *(*x *= 10, 12)	2*n *= 20	4*x*	a: GU289581, GU289582	Marcussen 682 (O)

*Viola mirabilis*	sect. *Viola *(*x *= 10, 12)	2*n *= 20	4*x*	a: GU289583, GU289584; b: GU289631	Marcussen 683 (O)

*Viola selkirkii*	sect. *Viola *(*x *= 10, 12)	2*n *= 24	4*x*	a: GU289589, GU289590; b: GU289634^b^	Marcussen 698 (O)

*Viola spathulata*	sect. *Viola *(*x *= 10, 12)	--	8*x*?	a: GU289593, GU289594, GU289595^b^, GU289596^b^, GU289597, GU289598; b: GU289636^b^	Marcussen 670 (O)

*Viola uliginosa*	sect. *Viola *(*x *= 10, 12)	2*n *= 20	4*x*	a: GU289585, GU289586; b: GU289632	Marcussen 662 (O)

*Viola verecunda*	sect. *Viola *(*x *= 10, 12)	2*n *= 24	4*x*	a: GU289591, GU289592; b: GU289633	Marcussen 697 (O)

*Allexis batangae*	Violaceae (outgroup)	--	2*x*	a: GU289562; b: GU289563^c^	Bos 4241 (UPS)

*Anchietea parvifolia*	Violaceae (outgroup)	--	2*x*	GU289559^c^	Myndel Pedersen 13944 (UPS)

*Corynostylis arborea*	Violaceae (outgroup)	--	2*x*	GU289560	Asplund 14509 (UPS)

*Cubelium concolor (= Hybanthus concolor)*	Violaceae (outgroup)	2*n *= 48	2*x*	GU289561	Pläck & Bodin s.n. (UPS)

*Hybanthus enneaspermus*	Violaceae (outgroup)	2*n *= 16, 32	2*x*	GU289558	unknown 2001-05-13 (UPS)

*Rinorea ilicifolia*	Violaceae (outgroup)	--	2*x*	GU289557	Friis et al. 2445 (UPS)

*Populus trichocarpa*	Salicaceae (outgroup)	2*n *= 38	--	a: DT509274; b: CV227572	--

*Manihot esculenta*	Euphorbiaceae (outgroup)	2*n *= 36, 54, 72	--	a: DV448133; b: (CK652029)	--

*Euphorbia esula*	Euphorbiaceae (outgroup)	2*n *= 20, 48-60	--	DV145650	--

Taxa used in this study, with respective GenBank accessions for DNA sequences and voucher information. For each taxon systematic affinity (sections within *Viola *for ingroup, and families within Malpighiales for outgroup), chromosome counts (2*n*, where available), and putative ploidal levels (*x*, inferred from the *NRPD2/E2 *data), are indicated. GenBank accession IDs are sorted by paralog (a and b), and by homoeolog (ascending numbers); gene copies excluded from phylogenetic analysis, because they were considered too short for reliable analysis, are put in brackets. Note that *NRPD2/E2-a *and *NRPD2/E2-b *are arbitrary labels, and that *NRPD2/E2-a *and *NRPD2/E2-b *in Euphorbiaceae, Salicaceae and Violaceae are not orthologous to each other. Herbarium acronyms for voucher specimen deposition (i.e., O, U) follow Holmgren and Holmgren [50].

^a ^the secondarily duplicated gene copies in *Viola canadensis *differed only in 3 (*NRPD2/E2-a*) and 8 (*NRPD2/E2-b*) substitutions, and their respective consensuses were used as single sequences in the analyses

^b ^partial sequence (exon 6 to exon 7); PCR 1 failed (see Figure 1)

^c ^partial sequence (exon 5 to intron 6); PCR 2 failed (see Figure 1)

**Figure 2:**
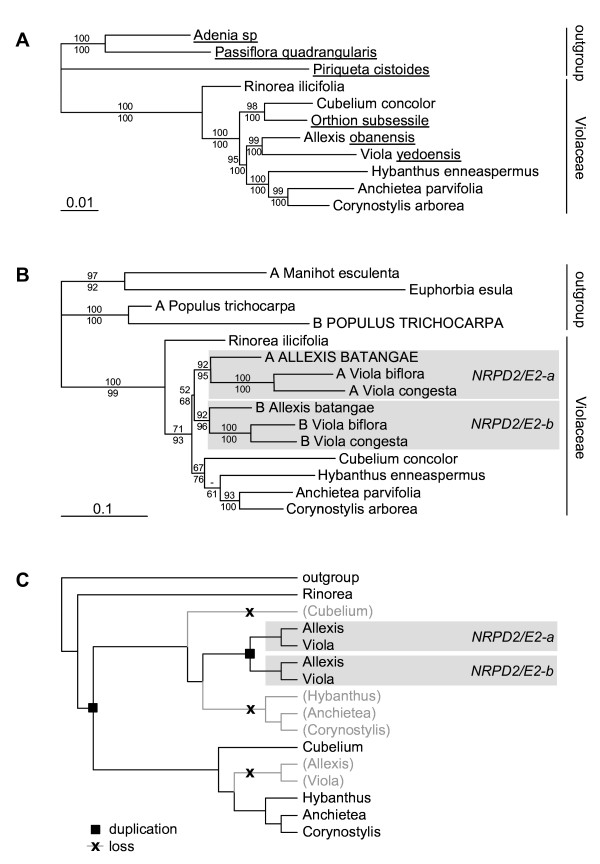
**Phylogeny of the *NRPD2/E2*-like gene family in Violaceae**. Bootstrap support values (1000 replicates) are indicated above (MP) and below (ML) branches, respectively. Names of pseudogenes are capitalized. A. Violaceae phylogeny inferred by ML analysis of the four genes *atp*B, *mat*K, *rbc*L and 16S. Sequence data were obtained from Tokuoka [25] with an additional *mat*K sequence of *Cubelium concolor *from GenBank (EF135550, as *Hybanthus concolor*). Names of taxa not included in phylogeny B are underlined. B. Phylogeny of *NRPD2/E2 *paralogs in Violaceae inferred by ML analysis. C. Tree reconciliation between the *NRPD2/E2 *gene tree (B) and the Violaceae organism tree (A), using the GeneTree software. The two inferred gene duplication events and three gene loss events are indicated.

Our inferences of the plastid and nuclear ribosomal phylogeny of Violaceae (Figure 2a) were congruent with previous analyses of the family, regarding both general topology [25] and the placement of *Cubelium *[34]. *Rinorea *was placed as sister to the rest of the Violaceae, with a *Cubelium *+* Orthion *clade and an *Allexis *+* Viola *clade as successive sisters to a *Hybanthus *(*Anchietea *+ *Corynostylis*) clade. All branches received high (95-100%) bootstrap support.

The *NRPD2/E2 *phylogenies (Figure 2b) were incongruent with the species tree. Again, *Rinorea *was placed as sister to the rest of the Violaceae with relatively high bootstrap support (MP: 71%/ML: 93%). Within rest-Violaceae three well-supported clades were found, one consisting of *NRPD2/E2-a *copy of *Allexis *and *Viola *(92%/95%), a second of the *NRPD2/E2-b *copy of *Allexis *and *Viola *(92%/96%), and a third (67%/76%) consisting of *Hybanthus *and *Cubelium *as sisters to a strongly supported (93%/100%) *Anchietea *+ *Corynostylis *clade. Whether it is *Hybanthus *(MP) or *Cubelium *(ML) that is sister to the rest within the last clade depends on the analysis, but neither topology receives strong bootstrap support (52% and 61%, respectively). Weak support is given for an *NRPD2/E2-a *+ *NRPD2/E2-b *clade (*Allexis *and *Viola*; 52%/68%). However, the inter-relationships of these three main clades remain elusive and depend on whether *Cubelium *and *Hybanthus *are included in the analysis (not shown).

No evidence of recombination was detected in the Violaceae alignment using GARD (see methods). Two possible recombination breakpoints were detected, but the topologies resulting from phylogenetic analyses of the partitions were congruent.

The reconciled tree (Figure 2c), constructed in GeneTree by embedding the *NRPD2/E2 *tree (Figure 2b) within the species tree (Figure 2a), explains the incongruence between these two trees by hypothesizing two events of gene duplication and three losses. A first duplication was postulated on the basal branch of all Violaceae except *Rinorea*, meaning that one paralog would have been lost in *Viola *and *Allexis *but retained in *Cubelium*, *Hybanthus*, *Anchietea *and *Corynostylis*. The second paralog may have been retained only in *Viola *and *Allexis*, before duplicating a second time in their common ancestor and diversify into their present *NRPD2/E2-a *and *NRPD2/E2-b *paralogs.

### *NRPD2/E2*-like homologs in Viola

There were considerable differences in the relative number of copies of *NRPD2/E2-a *and *NRPD2/E2-b *across lineages of the genus *Viola *(Table 1), but seen as a whole *NRPD2/E2 *always occurred in two or more potentially functional copies. Only the two diploid sections *Andinium *and *Chamaemelanium *appeared to have single and functional copies of each of *NRPD2/E2-a *and *NRPD2/E2-b*. All gene copies appeared functional in the neopolyploids of the latter section (*V*. *nuttallii *and *V*. *canadensis*). Non-functional gene copies were identified by the often numerous occurrence of premature stop codons and frameshift mutations within exons (up to a single 862 bp deletion comprising all of exon 6 in *B1*_*banksii*); in a single case (*NRPD2/E2-b *in *V*. *uliginosa*) the sequence was assumed to be non-functional because of a partial duplication within the highly conserved GEMERD amino acid motif of exon 7. Taxa of section *Erpetion *(*V*. *banksii*) and section *Chilenium *(*V*. *maculata*) had equal numbers of *NRPD2/E2-a *and *NRPD2/E2-b *copies; 5 and 4 of each, respectively, but differed in their respective numbers of putatively functional copies. All members of the sections *Melanium *and *Viola *had unbalanced numbers of *NRPD2/E2-a *and *NRPD2/E2-b*. Typically, taxa of section *Viola *had two putatively functional copies of *NRPD2/E2-a *and one non-functional copy of *NRPD2/E2-b *(except in *V*. *hirta *and in *V*. *spathulata*). Members of section *Melanium *had four to six copies of *NRPD2/E2-a*, of which one or several could be non-functional, but no copies of *NRPD2/E2-b*. Unbalanced numbers of *NRPD2/E2-a *and *NRPD2/E2-b *copies were found also in *V*. *brevistipulata *and *V*. *nuttallii *(section *Chamaemelanium*) but, in light of their ploidy levels and expected copy number, this likely reflects heterozygosity in one of the *NRPD2/E2-a *loci (*V*. *nuttallii*) or the *NRPD2/E2-b *locus (*V*. *brevistipulata*).

**Figure 3:**
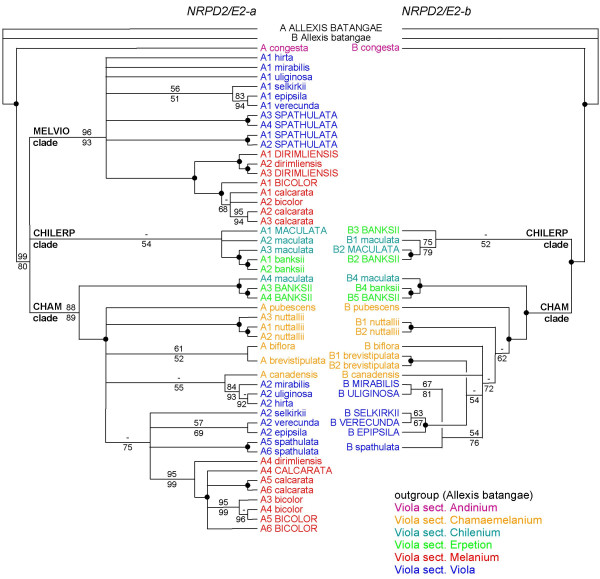
**Phylogenies of *NRPD2/E2-a *and *NRPD2/E2-b *in *Viola***. *Allexis batangae NRPD2/E2-a *and *NRPD2/E2-b *paralogs are used as outgroup. Bootstrap support values are indicated as percentages, MP values above branches (based on 1000 replicates), and ML values below branches (based on 100 replicates). Branches receiving high bootstrap support (>95% for both MP and ML) are indicated with a terminal dot. Pseudogenes are indicated in capital letters. Taxa are grouped and color-coded according to section.

The MP and ML phylogenies of *NRPD2/E2-a *and *NRPD2/E2-b *in *Viola *are all largely congruent (Figure 3) with an (as of yet) unpublished phylogeny for the genus based on another low-copy nuclear gene (Marcussen T, Oxelman B, Blaxland K, Jakobsen KS, in prep.), with the exceptions that *NRPD2/E2-b *is absent in the MELVIO clade, in the entire section *Melanium *and in *V*. *hirta *of section *Viola*. Generally higher bootstrap support was obtained for *NRPD2/E2-b *than for *NRPD2/E2-a*, reflecting that the former has ca 200 bp longer introns and therefore more phylogenetically informative sites. Working our way up from the root of the two consensus trees in Figure 3, *V*. *congesta *(section *Andinium*) is sister to the rest of the genus, sandwiched by branches receiving strong bootstrap support in all analyses. Next comes a polytomy of three lineages, here referred to as CHILERP, MELVIO (only *NRPD2/E2-a*) and CHAM. The CHILERP clade, which received only weak ML bootstrap support, but was recovered for both *NRPD2/E2-a *(54%) and *NRPD2/E2-b *(52%), consisted of various *V*. *banksii *(section *Erpetion*) and *V*. *maculata *(section *Chilenium*) lineages, of which one internal mixed species lineage received 100% bootstrap support. The MELVIO clade, missing for *NRPD2/E2-b*, received strong support for *NRPD2/E2-a *(MP: 96%/ML: 93%) and consisted of a basal polytomy of taxa of section *Viola *within which a strongly supported (100%) section *Melanium *is nested. The large and strongly supported CHAM clade included sequences from all represented sections except *Andinium *and, in the case of *NRPD2/E2-b*, *Melanium*. A basal dichotomy in the CHAM clade lead to two strongly supported sub-clades: one sub-clade consisting of one *V*. *maculata *sister to two *V*. *banksii *sequences, and a second sub-clade in which taxa of sections *Chamaemelanium *and *Viola *formed a polytomy; in *NRPD2/E2-a *section *Melanium *was a monophyletic group (92%/99%) within this basal polytomy. Within the polytomies of CHAM and MELVIO the species constellation of (*V*. *mirabilis *(*V*. *uliginosa *+ *V*. *hirta*), (*V*. *epipsila *+ *V*. *selkirkii *+ *V*. *verecunda*), and *V*. *dirimliensis *sister to the rest of section *Melanium *were common.

### Selective forces

**Figure 4:**
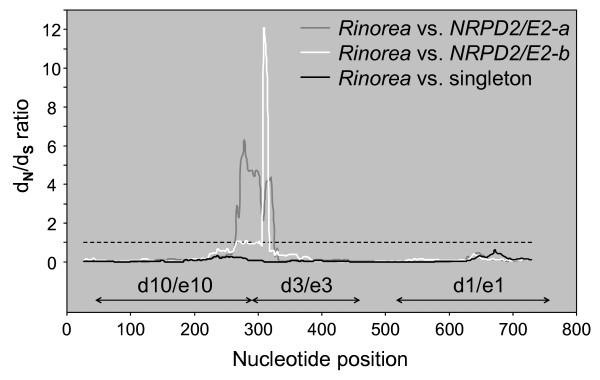
**Sliding window plot of *d*_*N*_/*d*_*S *_ratios for *NRPD2/E2 *in Violaceae**. The plot was generated by comparing the *Rinorea *sequence to singleton *NRPD2/E2 *in *Cubelium*, *Hybanthus *and *Corynostylis *(black), to *NRPD2/E2-a *in *Allexis *and *Viola *(gray), and to (3) *NRPD2/E2-b *in *Viola *(white). Window length was set to 54 bases and step size to 9 bases. Sites interacting with the other Pol IV/V subunits Nrpd1/Nrpe1 (d1/e1), Nrpd3/Nrpe3 (d3/e3) and Nrpd10/Nrpe10 (d10/e10) are shown, based on findings for Pol II [2]. Sites under neutral (*d*_*N*_/*d*_*S *_= 1) or positive selection (*d*_*N*_/*d*_*S *_> 1) are seen in a restricted 54 bp region, from position 249 through 302, for *NRPD2/E2-a *and *NRPD2/E2-b *while purifying selection (*d*_*N*_/*d*_*S *_< 1) predominates in the rest of the locus. *Corynostylis arborea *and *B_Allexis batangae *were excluded from the sliding window analysis because of a lack of data from exon 7.

The pattern of change of *d*_*N*_/*d*_*S *_ratios along the sequence is shown in Figure 4, using the sliding window option for pairwise comparison of *Rinorea *with three data sets: (1) 3 *NRPD2/E2 *sequences from *Cubelium*/*Corynostylis*/*Hybanthus*, in which the gene is not duplicated; (2) 13 *NRPD2/E2-a *sequences from *Allexis *and *Viola*; and (3) 10 *NRPD2/E2-b *sequences from *Viola*. The *d*_*N*_/*d*_*S *_ratios are well below 1 throughout most of the sequence for *Cubelium*/*Corynostylis*/*Hybanthus*, thus indicating purifying selection. It is for the most part also so for *NRPD2/E2-a *and *NRPD2/E2-b*, but for both paralogs a 54 bp (18 amino acid) region with *d*_*N*_/*d*_*S *_considerably higher than 1 is identified near the 3' end of exon 6 (nucleotide positions 249 through 302), indicating positive selection in both these paralogs. Within the region of positive selection a compensatory pattern conserving regionally the net charge of mutations was found (not shown): substitution of E/D (glutamic acid/aspartic acid) in position 300 is compensated for by gain of E in position 252 in *NRPD2/E2-b*. Thus, the positions of charged amino acids are subject to selection.

**Figure 5:**
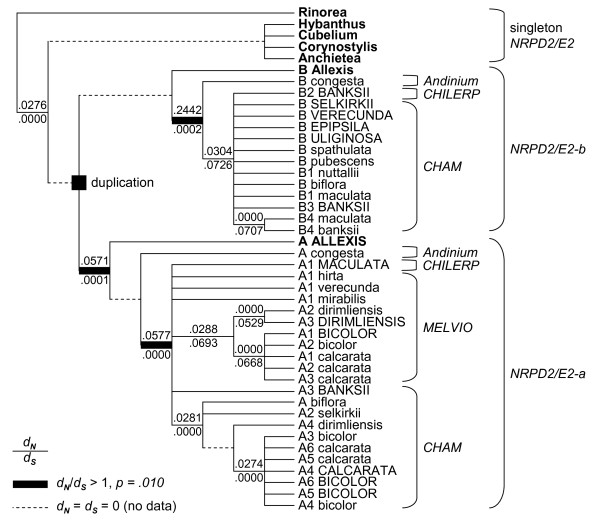
***d*_*N *_and *d*_*S *_estimated for *NRPD2/E2-a *and *NRPD2/E2-b *along individual branches in the Violaceae phylogeny**. Values of *d*_*N *_(above branches) and *d*_*S *_(below branches) along each branch were estimated by using the free-ratio model using the CodeML program in PAML [49]. For the branches drawn with bold lines, *d*_*N*_/*d*_*S*_was larger than 1 (*p *= 0.010) under a 2-rate model, which suggests that positive selection acted on these lineages. Branches collapsing in a MP phylogenetic analysis of the 54 bp region are indicated with broken lines. *NRPD2/E2 *duplication in the common ancestor of *Allexis *and *Viola *is indicated. Only species names are shown for *Viola*, and non-*Viola *taxon names are shown in boldface. Pseudogenes are indicated with capital letters.

**Figure 6:**
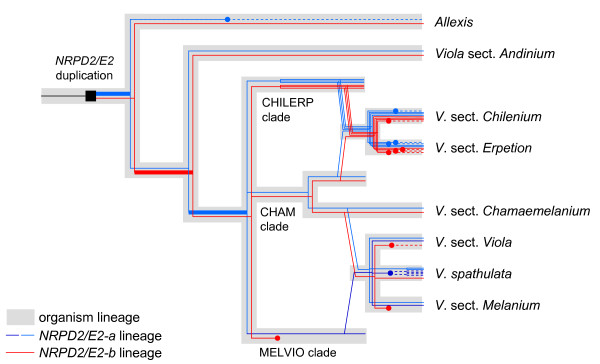
***NRPD2/E2 *gene lineage phylogeny versus organism phylogeny of *Allexis *and *Viola***. Unbroken lines denote putatively expressed paralogs, broken lines denote pseudogenes, and thick lines branches that have undergone positive selection (cf. Figure 5). Dots indicate events of pseudogenization or gene death. Truncated gray branches indicate the three (presumably extinct) lineages that contributed genomes to the four paleopolyploid *Viola *sections *Chilenium*, *Erpetion*, *Melanium *and *Viola*. Putative allopolyploidization events are indicated by diagonal *NRPD2/E2 *lines connecting the organism lineages. For simplificity, the sections *Melanium *and *Viola *are shown as derived from the same allopolyploidization event, and secondary gene duplications within section *Melanium *have been omitted.

The 54 bp region where positive selection was detected (Figure 4) was further analyzed using the CodeML software of the PAML package for estimating *d*_*N*_/*d*_*S *_ratios of 60 specified branches in the predefined phylogenetic tree. A 60-parameter model, assuming one *d*_*N*_/*d*_*S *_ratio for each branch, was found to marginally better fit the data (*p *= 0.0516) than a single-parameter model, assuming a uniform *d*_*N*_/*d*_*S *_ratio across all branches in the tree. Although many branches in the tree had positive *d*_*N*_/*d*_*S *_ratios, especially those immediately after the duplication basal to *Allexis *and *Viola*, only for the branch basal to *B_congesta *was the *d*_*N*_/*d*_*S *_ratio significantly larger than 1 (*p *= 0.0526). A model assuming a common *d*_*N*_/*d*_*S *_ratio for the three basal-most branches following the duplication, i.e. basal to *A_Allexis*, *A_congesta *and *B_congesta*, received strong support (*p *= 0.0101). Thus, both *NRPD2/E2 *paralogs seem to have been subjected to positive selection (*d*_*N *_>*d*_*S*_) soon after the duplication, but apparently not at exactly the same time (Figures 5 and 6). For *NRPD2/E2-a*, positive selection is hypothesized (i) immediately after the duplication of *NRPD2/E2 *and before the split of *Allexis *and *Viola*, and (ii) within the rest of *Viola *after *Viola *section *Andinium *split off, and finally (iii) also within the CHAM clade. For *NRPD2/E2-b *positive selection occurred somewhat later, and only in the branch leading to *Viola *(i.e. not in *Allexis*).

## Discussion

### *NRPD2/E2 *phylogeny within Violaceae

The *NRPD2/E2-a *and *NRPD2/E2-b *phylogenies differ in several respects from the already published organism phylogenies for *Viola *[26,32,35], based solely on the nuclear ITS region, and for Violaceae [25], based on 4 nuclear and chloroplast regions. Our results indicate that, at the family level, this incongruence is due to duplication of *NRPD2/E2 *and the uneven sorting of paralogs among lineages (Figure 2). Within *Viola*, the incongruence appears to result partly from the notorious failure of ITS to capture allopolyploid relationships, a common consequence of gene conversion among the often thousands of copies of this gene within the plant genome [33], and partly from the evolutionary unstable copy number of *NRPD2/E2 *(Figure 3). A genus phylogeny based on low-copy nuclear genes is currently being constructed and is largely congruent with the *NRPD2/E2-a *phylogeny (Marcussen T, Oxelman B, Blaxland K, Jakobsen KS, in prep.).

In comparison with an organism phylogeny of Violaceae, based on data from Tokuoka [25] with an extra accession of *Cubelium rbc*L, our data suggest that *NRPD2/E2 *was duplicated twice within the evolutionary history of the family. The reconciled GeneTree phylogeny (Figure 2C) indicated a first duplication relatively basally in the family, after the split of *Rinorea*, with subsequent complete sorting of paralogs in the descendant genera. so that one paralog was retained in *Cubelium*, *Hybanthus*, *Anchietea *and *Corynostylis*, and the second paralog was retained in *Allexis *and *Viola*. This interpretation, however, entirely rests on the conflicting phylogenetic position of *Cubelium *in the species phylogeny, which received strong bootstrap support (MP: 98%, ML: 100%), versus in the *NRPD2/E2 *phylogeny which was less strongly supported (MP: 67%, ML: 76%). On the other hand, we found no evidence that this incongruence resulted of recombination.

The second duplication event, in the common ancestor of the genera *Allexis *and *Viola*, is incontestable because it is retained in most of the descendants. Lineage sorting of paralogs may, however, also explain the phylogenetic pattern and link the two duplication events. The first duplication may in fact have persisted in the lineage leading to *Allexis *and *Viola*, but been subject to an event of sequence replacement in the common ancestor of these two genera. Either scenario would appear in the phylogeny as an independent duplication basal to *Allexis *and *Viola*.

Interestingly, for the Caryophyllaceae it has not been possible to trace back the origin of the *NRPD2/E2 *duplication event either. Judging by paralog similarity, the duplication in tribe *Sileneae *seems to be a relatively recent one and may well have occurred within this tribe [10]. In contrast, *Cerastium*, which belongs to another subfamily [36], has *NRPD2/E2-a *and *NRPD2/E2-b *paralogs that are substantially more divergent than in the *Sileneae *and that may result from an older duplication (Brysting AK, Mathiesen C, Marcussen T, in prep.). Thus, it may be that the small *NRPD2/E2 *gene family is subject to massive concerted evolution between paralogs, as already indicated in *Silene *by gene conversion (loss of *NRPD2/E2-b *and duplication of *NRPD2/E2-a*) within one lineage. Due to these factors it may be hard to pinpoint the duplication event on a phylogenetic tree.

Within the Violaceae, there are certain indications from ongoing research that the original duplication of *NRPD2/E2 *may be connected with whole genome duplications via allopolyploidy. Recent findings for the genus *Ionidium*, which belongs to the same clade as *Anchietea *and *Corynostylis *in the present study, based on karyology (Seo MN, Sanso AM, Xifreda CC, unpublished) and a low-copy nuclear gene (unpublished data), suggest that the currently accepted base chromosome number (*x *= 8) for this genus, and for large parts of the family, is in fact tetraploid. There is some evidence of paleaotetraploidy also in *Viola *as, apart from *NRPD2/E2*, also several other low-copy genes have been found to be duplicated, i.e. chalcone synthase [37], shikimate dehydrogenase (unpublished data) and homeotic floral genes (Ballard HE, personal communication).

Most *Viola *groups were found to have a more or less balanced number of *NRPD2/E2-a *and *NRPD2/E2-b *copies. Presumably due to redundancy following polyploidy, massive pseudogenization of this gene family has happened in the paleopolyploid sections *Chilenium *and *Erpetion*. However, the situation is very different in the other two polyploid sections, *Viola *and *Melanium*. These two sections have their allopolyploid origin in one or several wide hybridization events between two major diploid clades, CHAM and MELVIO. The CHAM clade, today represented by the diploid section *Chamaemelanium*, has apparently functional copies of both *NRPD2/E2-a *and *NRPD2/E2-b*, while the MELVIO clade, which is now extinct as diploid, has secondarily lost its *NRPD2/E2-b *paralog. Assuming subneofunctionalization of the two paralogs (which is suggested by positive selection, see below), this would mean that the remaining MELVIO paralog, which is by phylogenetic origin an "A" paralog, must have regained the ancestral expression state performing both "A" and "B" functions. Thus, the sections *Viola *and *Melanium *inherited one paralog of each *NRPD2/E2-a *and *NRPD2/E2-b *from the CHAM ancestor, and from the MELVIO an *NRPD2/E2-a *paralog with both "A" and "B" function. This "incomplete redundancy" in the polyploids may have lead to further gene death within the two sections. In species belonging to these sections (*V*. *spathulata *excepted), the current CHAM *NRPD2/E2-b *paralog is either a pseudogene (section *Viola *except *V*. *hirta*) or has been completely lost (section *Melanium *and *V*. *hirta*), while having two *NRPD2/E2-a *copies, one derived from CHAM and a second from MELVIO. In *V*. *spathulata *all the MELVIO paralogs have been pseudogenized and only the CHAM-derived paralogs are expressed; this was, apparently, also followed by a more recent polyploidization event.

Thus, both sections *Viola *and *Melanium*, although tetraploid, possess putatively functional *NRPD2/E2 *copies only of *NRPD2/E2-a*, derived from the ancestral MELVIO and CHAM genomes. As both these seem functional, and have not suffered the same fate as the *NRPD2/E2-b *paralog during the same period of time, it may be that some degree of *de novo *subfunctionalization has evolved between these two *NRPD2/E2-a *paralogs. Further research is needed to shed light upon this issue.

### Positive selection is seen in regions associated with subunit interaction

In cases with ongoing neofunctionalization following gene duplication, one would expect positive selection to be acting on parts of one or both paralogs, and *d*_*N *_to be larger than *d*_*S *_[e.g., [38]]. Our findings for *NRPD2/E2 *fit well with these assumptions: only purifying selection was detected among taxa with singleton *NRPD2/E2*; while soon after duplication of the gene in the common ancestor of *Allexis *and *Viola *both *NRPD2/E2 *paralogs seem to have been subjected to rapid sub- and neofunctionalization. This is detected as *d*_*N*_/*d*_*S *_ratios larger than 1 along these branches, indicating positive selection, especially early in the divergence process (Figure 6). This process appears to have happened at different times in *NRPD2/E2-a *and in *NRPD2/E2-b*. Our branch analysis suggests rapid specialization of *NRPD2/E2-a *in the common ancestor of *Allexis *and *Viola *while for *NRPD2/E2-b*, positive selection occurred at a later time and only in *Viola*, not in *Allexis*. Compared to *Viola*, higher redundancy in *Allexis *due to a still incomplete complementation of the two paralogs, may have facilitated pseudogenization of *NRPD2/E2-a *in *A*. *batangae*.

It is likely that the 54 bp region is important for the specialization and neofunctionalization of the two *NRPD2/E2 *paralogs in *Viola*. Crystallography of yeast Pol II has shown that this region of the second-largest subunit (Nrpb2) is part of a "hybrid binding" domain that is involved in subunit recognition and binding [2]. The first half of this region corresponds to an ordered loop interacting with the tenth-largest subunit (Nrpb10) and its second half forms an α-helix that interacts with the third-largest subunit (Nrpb3). Since structure and function tend to be conserved over all eukaryot Pols [2] and, especially, because of the close phylogenetic relationship between the Pol II and Pol IV/V subunit genes [3], we can assume that this region of Nrpd2/Nrpe2 interacts with homologs of Nrpd3 and Nrpd10. Under the assumption that the differentiation of *NRPD2/E2-a *and *NRPD2/E2-b *reflect specialization with respect to Pol IV and V, we suggest that this region is important for correct recognition of subunits specific to Pol IV and Pol V in *Viola*. This likely applies also to duplicated *NRPD2/E2 *in other eudicot lineages, and in this respect the between-paralog divergence of the very same region also in *Silene *[10] is noteworthy.

In *Arabidopsis thaliana *the exact subunit compositions of Pol IV and Pol V are known [1]. In this species, only a single copy of *NRPD2/E2 *is expressed (although another very similar duplicate is pseudogenized) and its protein product assembles with both Pol IV and Pol V [1]. Of the two subunit genes with whose gene products Nrpd2-a and Nrpd2-b interact, the *NRPD10 *homolog is not duplicated in *Arabidopsis *and shared between Pols II, IV and IV. *NRPD3/E3*, however, exists in two rather similar paralogs (85% sequence similarity at the protein level) that are incompletely subfunctionalized between Pol II/V and Pol V and apparently have been under positive selection (not shown). In the other genome sequenced eudicots, *Populus trichocarpa *and *Vitis vinifera*, neither of these genes are duplicated. If, however, *NRPD3/E3 *is duplicated in *Viola *and Violaceae, in addition to the basal differentiation of *NRPD1 *and *NRPE1*, this could give some indications about how *NRPD2/E2 *came to be duplicated in this angiosperm family.

## Conclusions

Aspects of the build, function and origin of the two atypical plant RNA polymerases Pol IV and Pol V are a hot topic in current research. This knowledge has in turn opened for study the dynamics of the origin and specialization of the individual subunits and their co-evolution within a phylogenetic framework.

Herein, we have presented the first documentation of possible co-evolution among subunits of Pol IV/V, from within the angiosperm family Violaceae. Following duplication, *NRPD2/E2-a *and *NRPD2/E2-b*, encoding Pol IV/V subunits, underwent rapid specialization (neofunctionalization) in a region that is important for subunit interaction and recognition. We conclude that correct recognition of the type-specific subunits is important for the correct function of each of Pol IV and Pol V.

Our study on Violaceae and previous studies on Caryophyllaceae draw a picture of *NRPD2/E2 *as a young gene family, still in the process of diverging and specializing and still subject to strong concerted evolution among paralogs. The few species and genera that have been studied show a variable number of Pol IV/V gene copies. Since the divergence of Pol IV and Pol V seems to have only barely preceded the radiation of the angiosperms, we can expect their young gene families to have acquired different lineage-specific specializations within angiosperms.

## Methods

### Material

The investigated 18 species of *Viola *(Table 1) were selected so as to cover the taxomomic diversity (i.e. following Ballard et al. [26]), geographical diversity and ploidal levels of the genus *Viola*. Represented in this study were *Viola *section *Andinium *(South America; *V*. *congesta*), section *Chilenium *(South America; *V*. *maculata*), section *Erpetion *(eastern Australia; *V*. *banksii*), section *Chamaemelanium *(mainly East Asia and North America; 4 species), section *Melanium *(mainly Mediterranean; 3 species) and section *Viola *(northern hemisphere; 7 species). Six outgroup taxa (Table 1) were selected from within Violaceae, of which *Allexis *was known to be phylogenetically close to *Viola *[25], and non-Violaceae outgroups from within the Malpighiales, *Populus trichocarpa *(Salicaceae), *Manihot esculenta *and *Euphorbia esula *(Euphorbiaceae).

### DNA isolation

DNA was extracted using a CTAB extraction protocol [39]. In most cases stock DNA was diluted 20 times for working solutions of which 1 μl was used per 20-40 μl PCR reaction. For "difficult" DNA preparations, where higher template amounts or cleaner template were needed in the PCR reaction, the obtained stock DNA solution was further cleaned using the DNeasy Blood & Tissue Kit (Qiagen, Düsseldorf, Germany), following the manufacturer's guidelines except that the first 2 steps were omitted; the obtained working solution was not diluted, and 5-10 μl were used in 80-160 μl PCR reactions divided into an appropriate number of tubes.

### PCR and sequencing

Primer sequences and standard PCR conditions are presented in Table 2. The nomenclature of exons and introns follows the terminology in *Arabidopsis*. The *NRPD2/E2 *locus, ranging from exon 5 through most of exon 7, was in most taxa amplified in two PCRs with overlapping range, one PCR covering exon 5 through intron 6 using the primers 5F2898 and Svex7R3420, and a second covering exon 6 through exon 7 using the primers vex6F3263 and 7R3883 (Figure 1). This approach was preferred to amplifying the entire locus in a single PCR, because (1) it increases the chance of discovering all paralogs (especially for pseudogenes where the primer binding sites are no longer conserved), because (2) it reduces the amount of PCR recombination which is expected to increase with gene copy number, their similarity and length of the amplified fragment [cf. [40]]. Where one of the PCRs failed (notably for the outgroup taxa for which DNA was extracted from herbarium material and of inferior quality), shorter stretches of DNA were sought amplified in three separate PCRs, (1) using the primer pairs 5F2898 (or 5F3062) and vex6R3371, (2) vex6F3263 and Svex7R3420, and (3) vex7F3418 and 7R3883. The primers were designed based on DNA sequences available on GenBank, and in some cases based on already existing *Viola *sequence data.

**Table 2:** Standard PCR and sequencing primers, primer combinations and annealing temperatures used

Region	Forward primer	Reverse primer	Annealing temperature
ex5-in6 (PCR 1)	5F2898: TTGACAGCCTYGATGATGAT	Svex7R3420: ATCTTGAAAATCCAGCCC	52°C
ex5-in6	5F3062: AATGATGASGGGAAGAATTTTGC	Svex7R3420	52°C
ex6-ex7 (PCR 2)	vex6F3263: GYCARCTYCTTGAGGCTGC	7R3883: ATVCCCATGCTGAAKAGCTCYTG	59°C
ex5-ex6	5F2898	vex6R3371: YMTCRACACTGGGAGTGGAG	54°C
ex5-ex6	5F3062	vex6R3371	57°C
ex6-in6	vex6F3263	Svex7R3420	52°C
ex7	vex7F3418: GGCTGGATTTTCAAGATGG	7R3883	55°C

PCR mix: 20 to 40 μl reactions; 0.2 mM dNTPs, 0.25 μM of each of the primers, 1× Phusion HF buffer, 0.008 U/μl Phusion polymerase. The PCR conditions were as follows: initial denaturation at 95°C for 30 s followed by 35 cycles of 95°C for 9 s, annealing at a temperature specified below for 30 s, and 72°C for 30 s. The PCR ended with 7:30 minutes at 72°C and subsequent soak at 10°C.

PCR products were separated by electrophoresis on 1% agarose gels, and multiple bands were cut out separately and cleaned using the E.Z.N.A. Gel Extraction Kit (Omega Bio Tek, Doraville, GA, USA) following the manufacturer's manual. Some cleaned products were sequenced directly, but generally these were cloned using the TOPO TA Cloning Kit (Invitrogen, Carlsbad, CA, USA) according to the manufacturer's manual, with the exception that only half of the volumes recommended for the reactions were used. Between 3 and 20 positive colonies from each reaction were screened by direct PCR using primers TOPO_F (GGCTCGTATGTTGTGTGGAATTGTG) and TOPO_R (AGTCACGACGTTGTAAAACGACGG). PCR products were diluted 10 times and sequenced one way using either T7 or M13R universal primer. Sequencing was done with BigDye 3.1 sequencing Kit (Applied Biosystems, Foster City, CA, USA) on 3730 ABI DNA analyzer (Applied Biosystems).

All sequence chromatograms were controlled manually and sequence alignments established in BioEdit [41] by manual adjustments. Indel characters were coded by using the simple gap-coding method in SeqState [42] and appended to the alignment. Four data alignment matrices were generated; these are available as additional files 1, 2, 3 and 4. (1) The first (Violaceae) matrix (see Additional file 1) consisted of intron and exon sequences of *Allexis batangae*, *Anchietea parvifolia*, *Corynostylis arborea*, *Cubelium concolor*, *Hybanthus enneaspermus*, *Rinorea ilicifolia*, *Viola biflora *and *V*. *congesta *aligned to an outgroup consisting of GenBank exon-only sequences of non-Violaceae *Populus trichocarpa*, *Manihot esculenta *and *Euphorbia esula*. (2) The second (*Viola*) matrix (see Additional file 2) consisted of all *Viola *sequences aligned to *Allexis batangae *sequences (outgroup). (3) The third matrix (see Additional file 3) was used to examine *d*_*N*_/*d*_*S *_ratios with a sliding window approach using the DnaSP software, and consisted of only exon sequences of all the non-*Viola *Violaceae sequences along with all *NRPD2/E2-a *and *NRPD2/E2-b *sequences of *V*. *congesta*, *V*. *banksii*, *V*. *maculata*, *V*. *biflora*, *V*. *brevistipulata*, *V*. *nuttallii *and *V*. *pubescens*. (4) The fourth matrix (see Additional file 4) was used to estimate individual *d*_*N*_/*d*_*S *_ratios for phylogenetic branches using the PAML software; this matrix consisted of a reduced data set of the 44 Violaceae taxa having unidentical *NRPD2/E2 *sequences (i.e., duplicate sequences were removed) within a 54 bp exon domain in which positive selection was detected in the DnaSP analysis. *Rinorea *was used as outgroup.

### Phylogenetic reconstruction

The Violaceae and *Viola *matrices were used for phylogenetic reconstruction. Owing to a large number of sequences in the *Viola *matrix *NRPD2/E2-a *and *NRPD2/E2-b *were analyzed separately, using maximum parsimony (MP) and maximum likelihood (ML). MP analyses of all three data sets were performed with TNT version 1.1 [43], using traditional search, tree bisection-reconnection (TBR) branch swapping, 10 replicates (number of added sequences), and 10 trees saved per replication. Maximum Parsimony bootstrap analyses were carried out with the same settings and with 1000 replicates. Maximum Likelihood analyses of all three data sets were performed with Treefinder version of March 2008 [44] and run with different nucleotide substitution models for the three partitions of the sequence data: exons, introns and coded indels. Nucleotide substitution models for the exon and intron partitions were proposed by Treefinder, while for coded indels a uniform rate model (Jukes-Cantor) was applied (in Treefinder this substitution model had to be specified as "HKY [{1,1,1,1,1,1}, Optimum]:GI [Optimum]:4"). The Violaceae matrix (1) was analyzed using the 4-rate model J1+I+G (TA = TG; CA = CG) for both exons and introns. The *Viola *matrix (2) was analyzed with the 3-rate model TN (TA = TG = CA = CG) for exons and the 4-rate model J1+I+G (TA = TG; CA = CG) for introns. Mamimum Likelihood bootstrap analyses were carried out with the same settings and with 100 replicates.

### Detection of gene duplication in Violaceae

The obtained *NRPD2/E2 *phylogeny was incongruent with the organism phylogeny of Violaceae, and so we further analyzed the Violaceae matrix for possible gene recombination and/or duplication events. The GeneTree software [45] was used to construct a reconciled tree by embedding the *NRPD2/E2 *tree within the Violaceae species tree. The Violaceae species tree was obtained from re-analysis of Tokuoka's [25] 4-gene data set of *atp*B, *mat*K, *rbc*L and 16S for a taxon subset corresponding to the one used in this study. *Cubelium concolor *was not included in Tokuoka's phylogeny but a *mat*K sequence was available on GenBank (EF135550, as *Hybanthus concolor*). In order to firmly place *Cubelium *in the family phylogeny and to compensate for weaker data for this taxon, we also included in our analysis *Orthion subsessile*, which has been considered close to *Cubelium *on morphological grounds [34]. Maximum Parsimony and ML analyses were carried out as above, except for ML using a 6-rate substitution model GTR+G. To screen the Violaceae alignment for possible recombination we employed the Genetic Algorithms for Recombination Detection (GARD) [46,47], on the exons only, using general parameter settings (GTR model of nucleotide substitution and beta-gamma rate variation with 2 rate classes). We then separated the alignments at the detected breakpoints within the region, estimated MP phylogenetic trees (1000 bootstrap replicates) for the individual sections, and checked for incongruent topologies.

### Detection of positive selection

Estimating *d*_*N *_(the number of nonsynonymous substitutions per nonsynonymous site) and *d*_*S *_(the number of synonymous substitutions per synonymous site) between coding sequences is useful for detecting whether there has been purifying selection (*d*_*N*_/*d*_*S *_< 1), neutral evolution (*d*_*N*_/*d*_*S *_= 1) or positive selection (*d*_*N*_/*d*_*S *_> 1).

For data matrix 3 (exons), the polymorphism and divergence module of the DnaSP package [48], using the sliding window option, were used to make a graphic representation of the pattern of change of *d*_*N*_/*d*_*S *_ratios along the sequence. We generated three sequence categories that were each compared to *Rinorea *(which is sister to the other examined Violaceae taxa). The first category consisted of all taxa (except *Rinorea*) having singleton *NRPD2/E2*, i.e. *Corynostylis*, *Cubelium *and *Hybanthus*. The second category consisted of 13 *NRPD2/E2-a *exon sequences from *Allexis *and *Viola *(*V*. *congesta*, 2 of *V*. *banksii*, 3 of *V*. *maculata*, *V*. *biflora*, *V*. *brevistipulata*, 3 of *V*. *nuttallii *and *V*. *pubescens*). The third category consisted of 10 *NRPD2/E2-b *exon sequences from the same *Viola *taxa as for the second category. As DnaSP does not support gaps nor stop codons, pseudogenes (except for *Allexis NRPD2/E2-a*, because of its phylogenetic position) and incomplete sequences (e.g., *Anchietea NRPD2/E2 *and *Allexis NRPD2/E2-b*) had to be omitted. Within *Viola*, taxa of the sections *Melanium *and *Viola *were omitted because they do not possess functional copies of both *NRPD2/E2-a *and *NRPD2/E2-b*.

For the 54 bp region for which positive selection was detected with DnaSP, we used the CodeML software of the PAML package [49] to further determine at which branches in the phylogeny positive selection had occurred. A simplified organism phylogeny was used as input tree file due to the short length (54 bp) of the sequence analyzed (Figure 5).

## Authors' contributions

TM designed the study, collected material, contributed the molecular studies, performed the phylogenetic and selection analysis and led the writing of the manuscript. AS contributed to the molecular studies. BO and KSJ participated in the coordination and design of the study, interpretation of the results and writing of the manuscript. All authors read and approved the final manuscript.

## Supplementary Material

Additional file 1 **Violaceae matrix**. This file represents the *NRPD2/E2 *alignment from 8 Violaceae taxa aligned to exon sequences of a non-Violaceae outgroup. Click here for file

Additional file 2 ***Viola *matrix**. This file represents the alignment of *NRPD2/E2 *copies from 18 *Viola *taxa aligned to *Allexis batangae *as outgroup. Click here for file

Additional file 3 **DnaSP Positive selection sliding window matrix**. This file represents the alignment of 27 *NRPD2/E2 *exon sequences from Violaceae taxa used to examine *d*_*N*_/*d*_*S *_ratios with a sliding window approach using the DnaSP software. Click here for file

Additional file 4 **PAML positive selection matrix**. This file represents the 54 basepair alignment of *NRPD2/E2 *exon sequences for 44 Violaceae taxa. Click here for file
